# At home in the margins

**Published:** 2016-04-14

**Authors:** Surita Parashar

**Keywords:** homelessness, HIV and AIDS, Photovoice, marginalized populations, participatory action research

## Abstract

This photo essay explores what constitutes ‘home’ for people living with HIV in Vancouver’s Downtown Eastside. It is a collaboration between myself and eight community researchers – four women and four men – who are living with HIV and have experienced significant periods of homelessness in this context. Over the course of four weeks in 2011, the community researchers were trained in Photovoice methods and took photographs of their homes and neighbourhoods using disposable film cameras.

‘The poorest postal code in Canada’. This is perhaps the most common phrase associated with Vancouver’s Downtown Eastside, an inner-city neighbourhood characterized by an open drug scene, high levels of poverty and unemployment, and high rates of homelessness. Those who live and dwell in the Downtown Eastside are similarly cast as ‘vulnerable’ (at best) or ‘junkies’ (at worst), bearing the stigma associated with poverty, disease, addiction, mental health disorders, and crime (Knight 2014). Central to narratives of the Downtown Eastside and its residents are its single-room-occupancy hotels (SROs). These hotels, some of which are one hundred years old, were originally built to meet the lodging needs of Vancouver’s seasonal workers. Today, many SROs have been purchased by the city and converted into low-barrier social housing run by various agencies. Other buildings continue to be owned and operated by private ‘slumlords’. Rooms average one hundred square feet in size, and include a sink. Toilets, showers, and cooking facilities are typically shared. Many SROs are unsanitary and infested with bugs – conditions that have been reported on extensively in the media (McElroy 2015; Austin 2015; Lupik 2014). Thus, while SROs do provide shelter from the elements, a lack of access to clean water, sanitation, and safety means that many inhabitants continue to live in ‘relative homelessness’.

The photos taken by the community researchers illustrate what it means to live each day ‘on the edge of homelessness’ (Swanson and Herman 2014), but they also reflect the ways in which people actively create home in places like the Downtown Eastside (Robertson 2007). Importantly, the images, and the narratives they evoked amongst community researchers in group and one-on-one debrief sessions with me, convey how a sense of being ‘at home’ is not always tied to the presence or absence of material shelter, but rather to social connection, routine, personal dignity, pride, and sense of belonging.

Photographs of bugs, rodents, and cockroaches are powerful symbols of the marginalization of those who are forced to dwell alongside them, echoing what Robertson (2007, 528) calls ‘the conflation of persons and place occurring in stigmatized space’. Randy would often talk about his run-down hotel as ‘the best he could do’, or as a lot that he ‘deserved’, conveying the ways in which some Downtown Eastside residents internalize dominant narratives about SROs and their inhabitants. Simultaneously, however, people in the group spoke about creating a ‘sense of home’ amidst substandard living conditions. Home making occurred both within and outside of SROs. Randy showed me a photo he had taken of graffiti painted on the side of his building, explaining, ‘We don’t have a lot of flowers where I live so we painted some on the wall’. The community researchers identified home as an important outlet for ‘self-expression’ – a place that one controls and defines as they like. The mural on the side of an otherwise dingy SRO was a gesture of home making that projected beauty, community, and pride in one’s neighbourhood onto the urban landscape, countering popular imagery of the Downtown Eastside as a dilapidated, neglected, urban ghetto. Community researchers also ‘found home’ in outdoor spaces such as gardens, parks, and patches of greenery throughout the downtown core. In these ‘normal’ spaces it was possible to meet and socialize with partners, children, and friends – visitor policies at SROs are often highly restrictive – allowing people to create or find a sense of home in the neighbourhood.

Randy talked extensively about his efforts to bring order to the disorder of his room at the SRO, which was infested with rodents. Looking at a photo he had taken of the area underneath his sink, which was covered in mouse droppings that he continually attempted to address with various cleaning and exterminating products (to no avail – the mice were relentless), Randy explained, ‘You don’t see that – in the closets here – that all my clothes are hung up, and that up in the drawer there – you know, my socks and stuff are all put away nice and neat and everything, and my bed’s made and – you don’t see that part’. By pointing to what is outside of the frame of his image, Randy drew attention to the ways in which people living in the margins assert control over their surroundings. What appear to be mundane objects and routines – a made bed and folded socks – are important gestures of home making through which residents challenged dominant narratives about SROs and their inhabitants. As he had at many points in our conversation, Randy turned back to the photo and said, with a mix of anger and melancholy, ‘You just see this shit that is there – and it’s never going to go away’.

At the start of the project, four of the eight community researchers had recently found a more permanent place to call home. After spending several years living in run-down SROs and alleyways in the Downtown Eastside, Dan received a portable housing subsidy from the government and a local social housing agency dedicated to supporting people living with HIV. The subsidy allowed Dan to secure a one-bedroom apartment in Richmond, a suburb of Greater Vancouver, giving him both geographical and social distance from his former life in the Downtown Eastside.

To illustrate how drastically his life had changed with the support of the housing subsidy, Dan created a posed photograph of several symbolic items, including a piece of paper on which he had drawn two columns labelled ‘unstable’ and ‘stable’. The word ‘welfare’ was scrawled in the ‘unstable’ column, under which lay a crumpled envelope that once held a social assistance cheque. As he pointed out the envelope in the photo, Dan explained, ‘Without support [the housing subsidy] I was unemployed, on welfare’. He gestured to a pile of used cigarette butts and bus tickets picked up off the street and continued, ‘[I was] picking up cigarette butts, because [I had] no money. I used to have such a hard time before, because I wouldn’t even get a bus pass. So I’d have to find used bus tickets and [be given] a hard time by the bus driver’.

Dan then turned his attention towards the column labelled ‘stable’ and said proudly, ‘Instead of that unemployment cheque or welfare cheque, it’s [an] actual paycheque. I managed to go back to work’. Dan explained that the transition to stable housing meant that he was no longer worried about where he was going to sleep, or how he was going to keep his belongings safe, which freed up his time and energy to find a job. He continued, ‘Now I’ve got money, I can buy my own smokes. Hold my head up a little bit higher. Don’t have to show the whole world that I’m a bum’.

At the bottom of Dan’s image is a scale. Weight change related to housing stability was an important theme highlighted by many community researchers. Wasting – unintended and progressive weight loss – is a common complication of HIV, and something that all of the group members had experienced. Weight gain is one of the immediate signs of improvement for people living with HIV who start or resume treatment. Dan had placed a piece of paper on the scale with two numbers associated with his ‘stable’ and ‘unstable’ columns. He recalled that when he was living in SROs and on the streets he got down to 140 pounds. After moving into more stable housing, his weight rose to 170 pounds. ‘My health’s come back’, he reflected.

Dan’s juxtaposition of life with support and without support resonated with all members of the group, whether through their own experiences or their aspirations. The items displayed in his photo – a bus pass, a full pack of cigarettes, a pay stub – were markers of not just of greater stability and improved health, but of personal dignity. Dan’s image is a powerful illustration of how one’s housing situation is intimately linked to a sense of pride, self-esteem, belonging, and social well-being (Robertson 2007).

Home is imbued with meaning far beyond its role in providing shelter (Mallett 2004); ‘being-at-home’ may not be grounded in a single site, but allude to one’s well-being as it is connected to a variety of spaces, including neighbourhoods and cities. These community researchers’ narratives highlight the ‘diverse ways people “do” and feel home’ (Leith 2006) and how a ‘sense of home’ – whether felt, created, or found – positively impacts physical and mental health. Alongside advocating for sustainable affordable housing, this project underscores the need to cultivate spaces in which individuals who are marginalized by social structural inequities can be ‘at home’ when they do not have a home of their own.

## Figures and Tables

**1:**
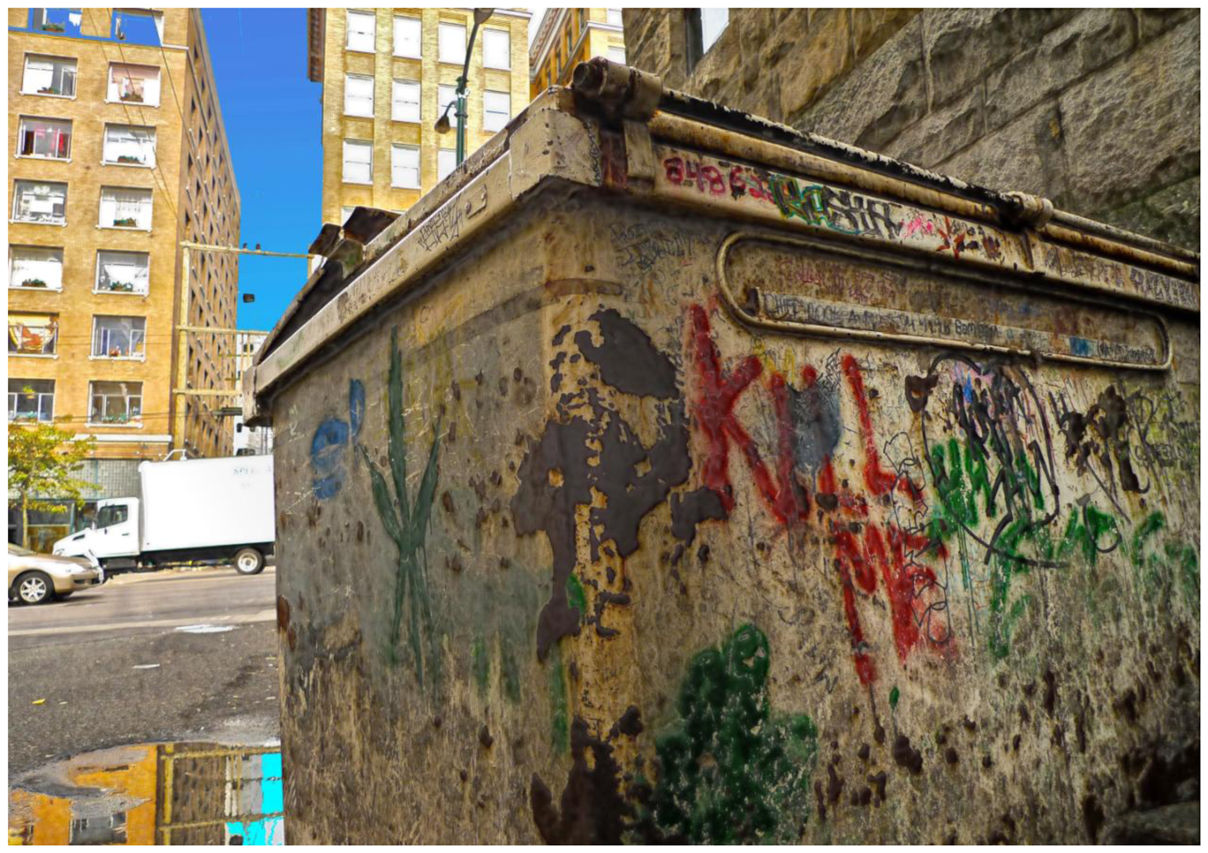
Eastside back alley – Rob

**2:**
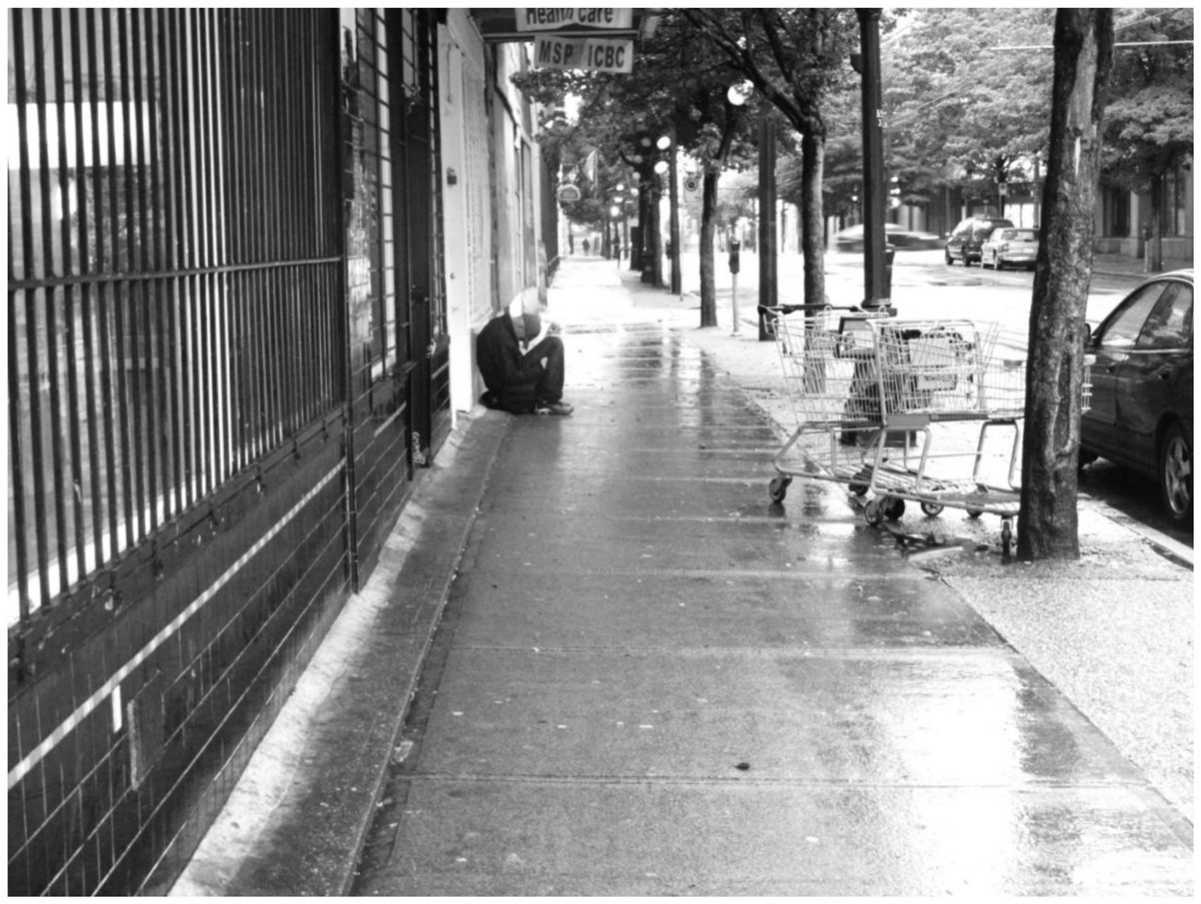
In need of mercy – Valerie

**3:**
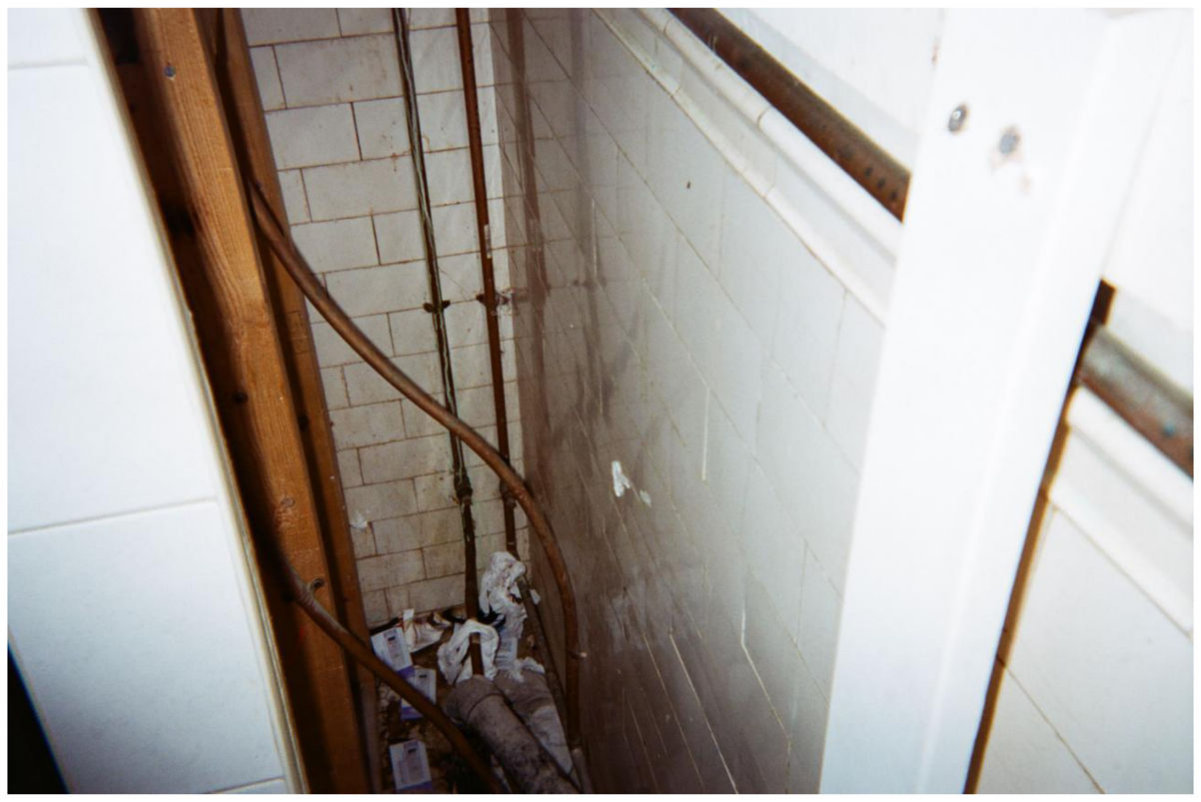
The bathroom shared by seventy residents – Lora

**4:**
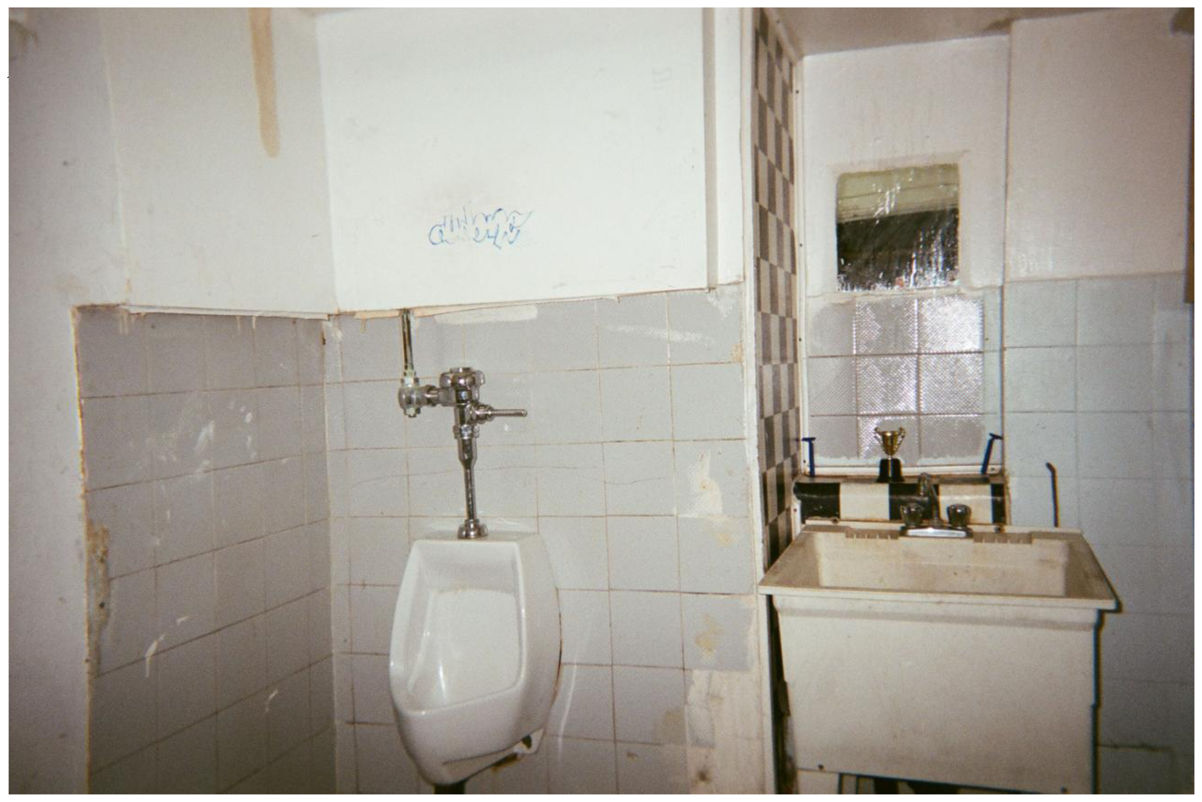
This is where I had to wash my hands and dishes – Valerie

**5:**
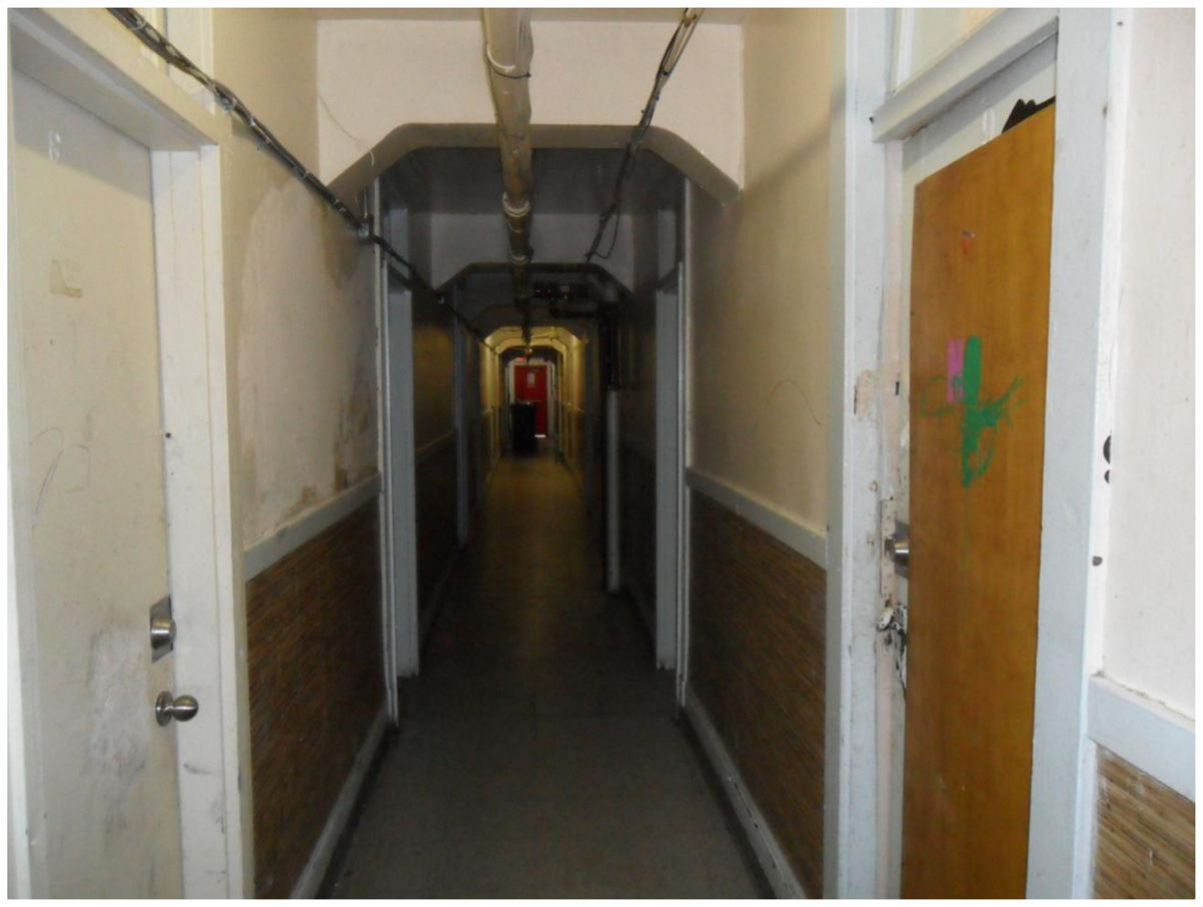
Broken lights, broken locks, broken hope – Valerie

**6:**
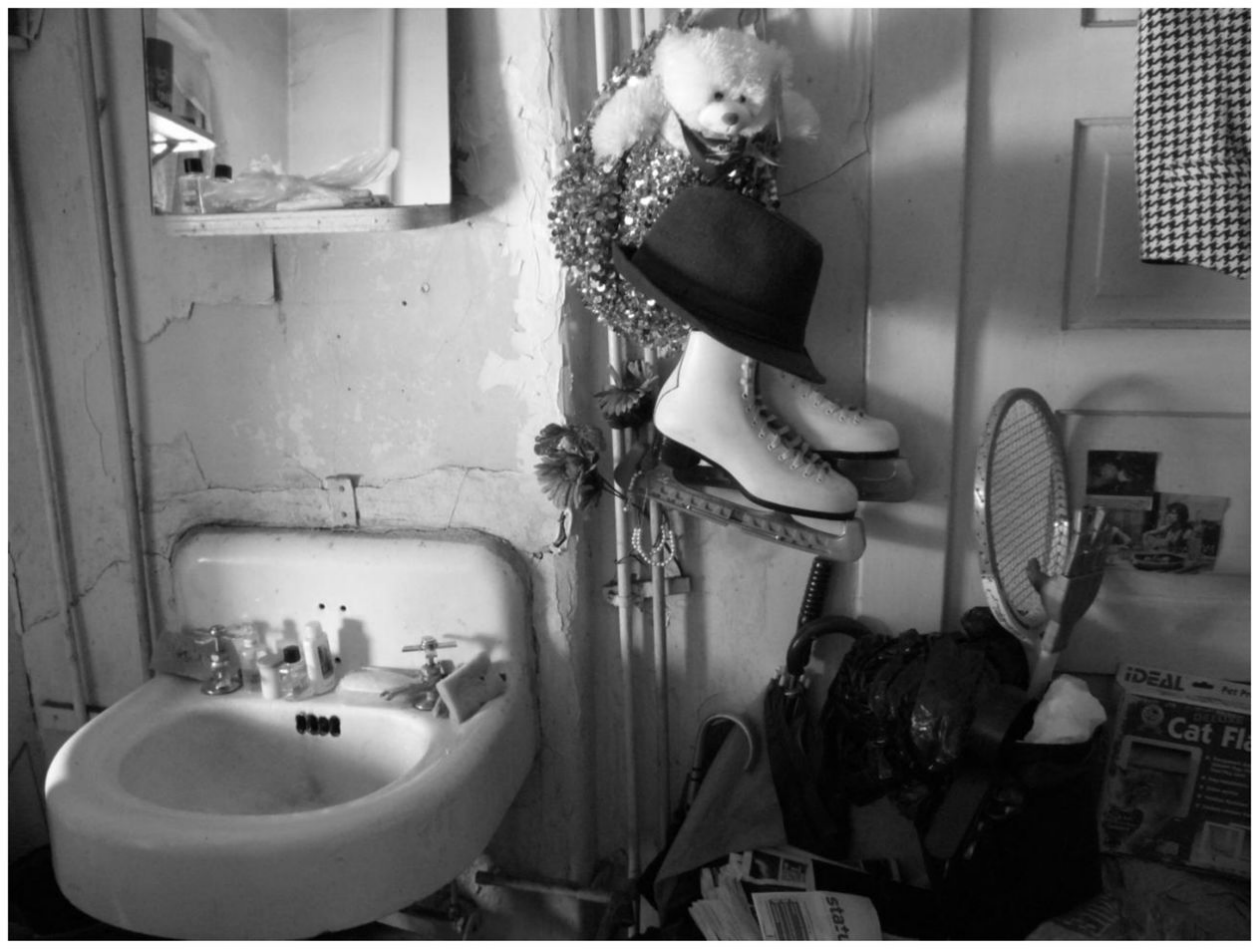
In the middle of all this hoarding, a bit of hope, a flower – Jenny

**7:**
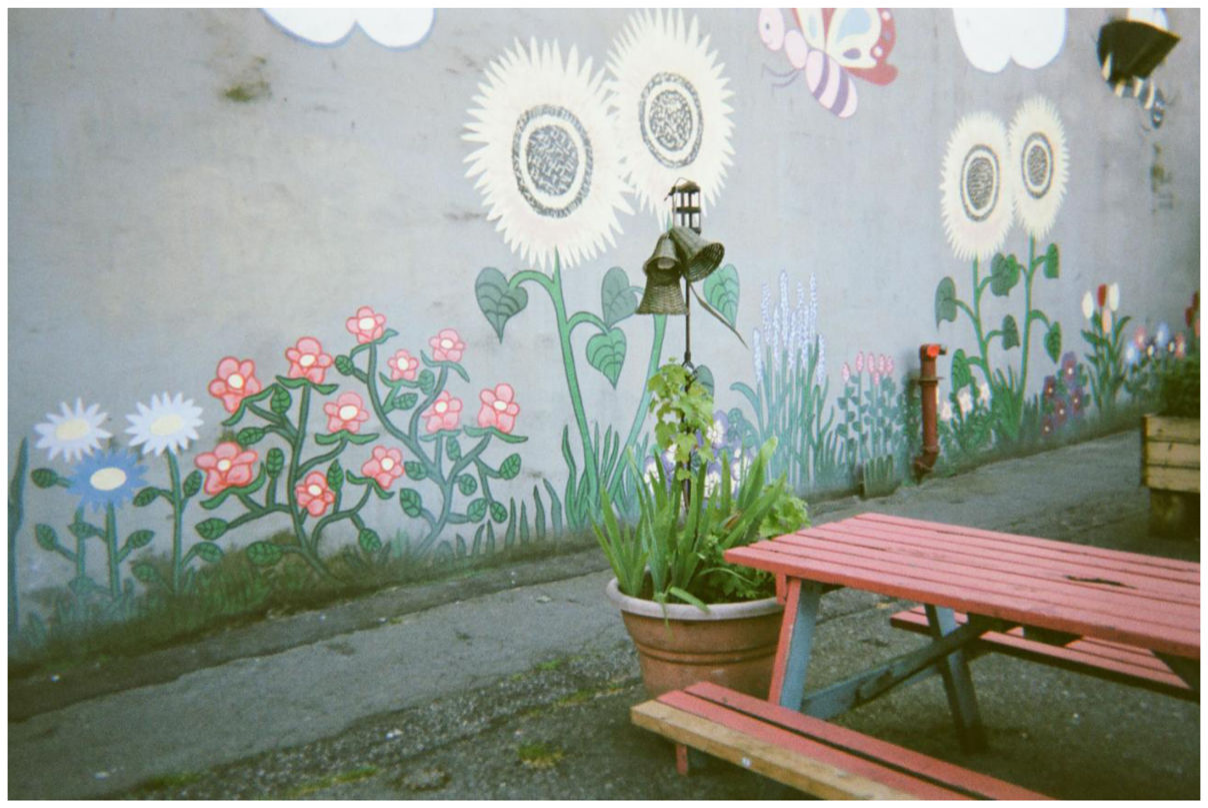
We don’t have a lot of flowers where I live so we painted some on the wall – Randy

**8:**
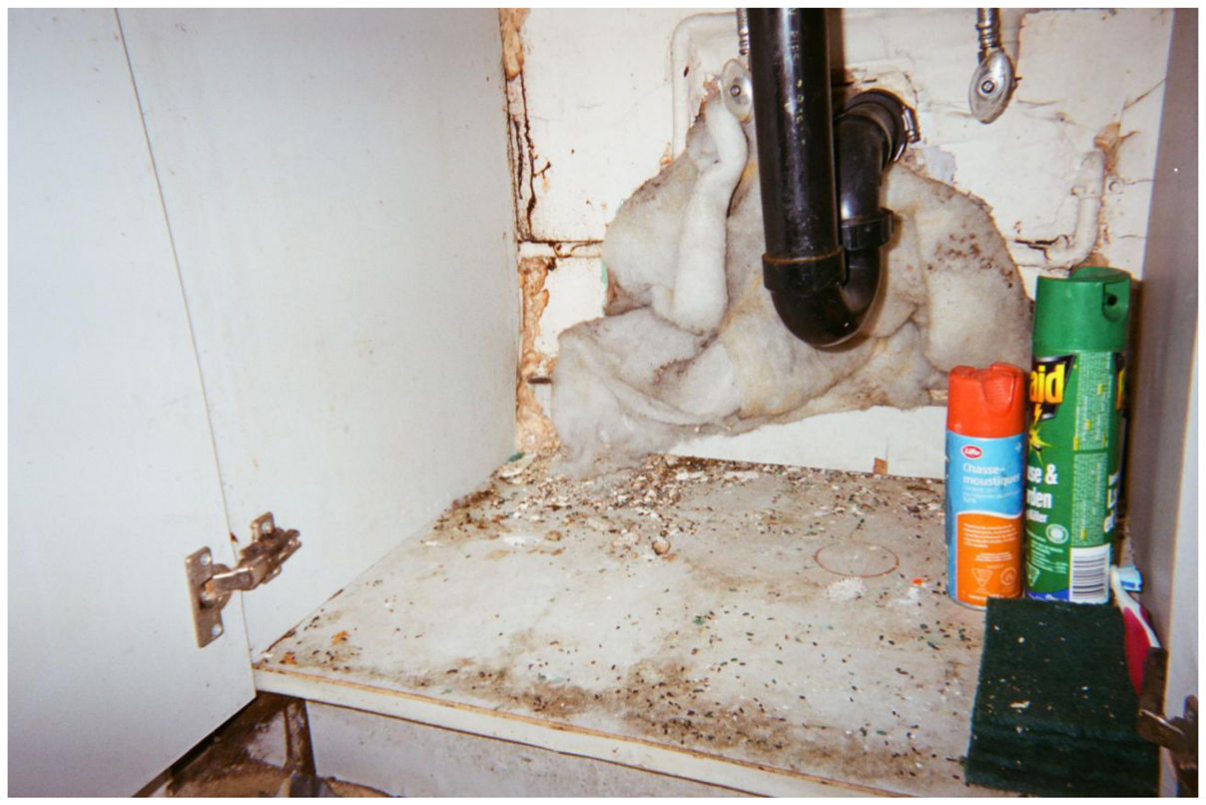
Under my sink live some mice – Randy

**9:**
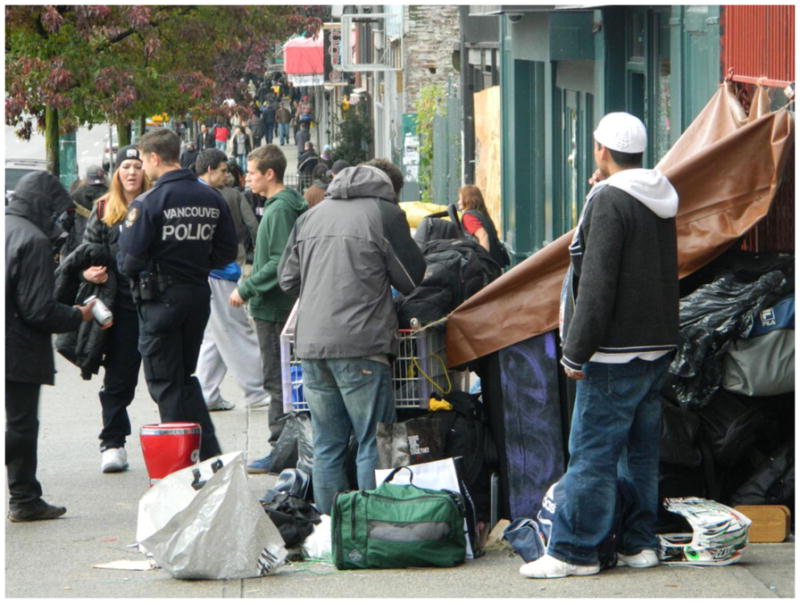
Where do I go if I can’t be here? – Valerie

**10:**
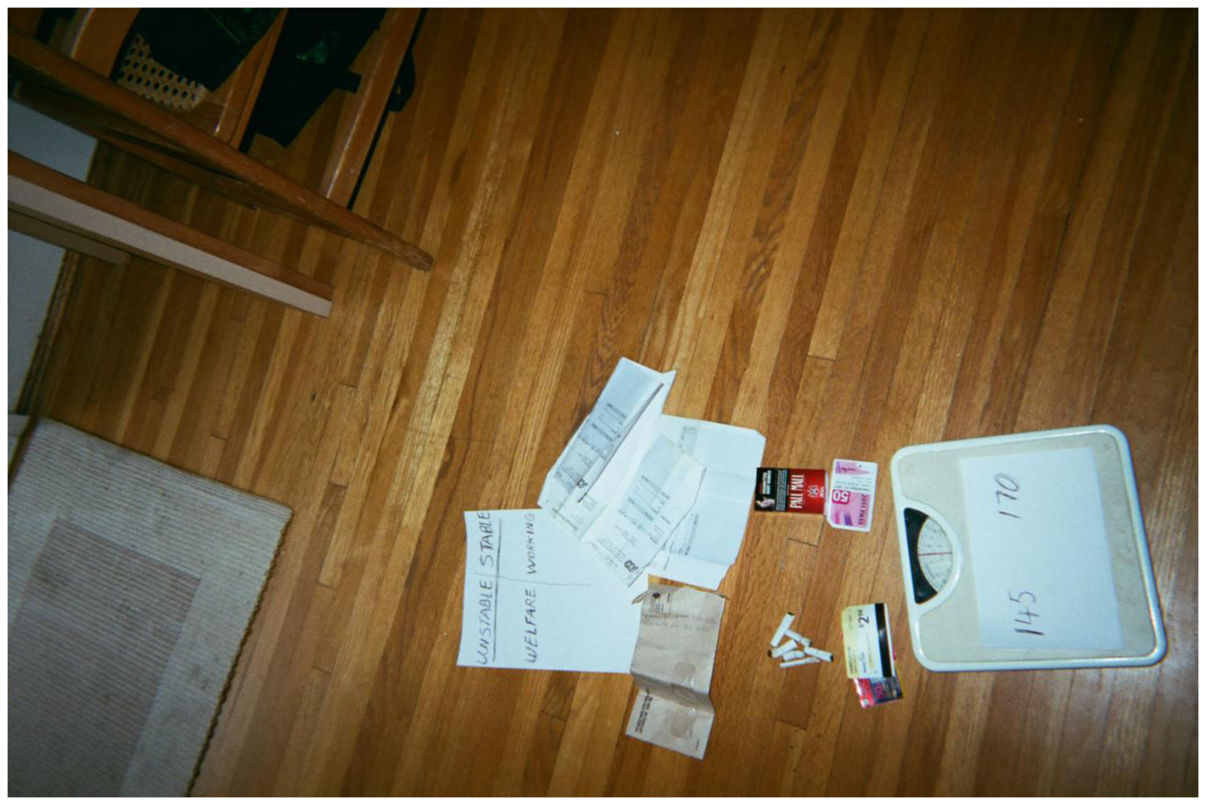
With support and without support – Dan

**11:**
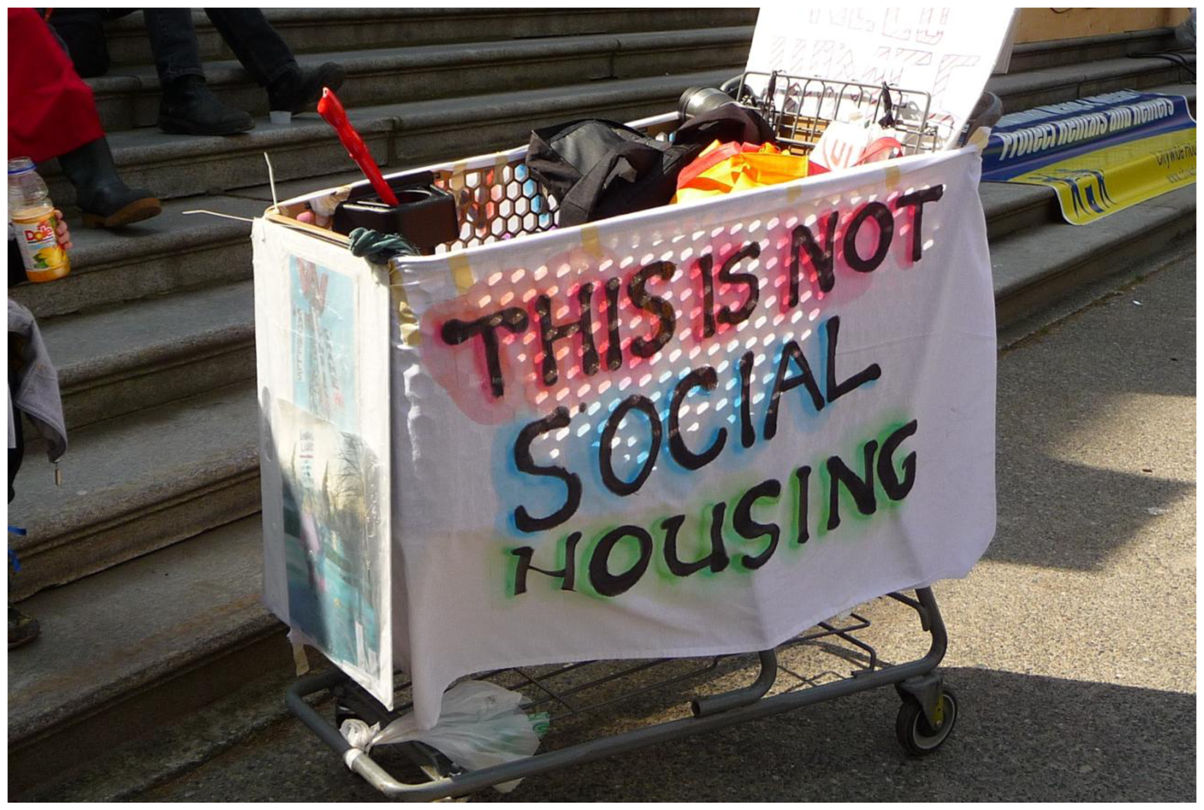
This is not social housing – Rob

## References

[R1] Austin Ian (2015). Vancouver Tenants File Court Challenge against Downtown Eastside SRO Landlords. Vancouver Sun.

[R2] Knight Leo (2014). Money Wasted on Vancouver’s DTES. 24 hours Vancouver.

[R3] Leith Katherine H (2006). “Home Is Where the Heart Is…Or Is It?”: A Phenomenological Exploration of the Meaning of Home for Older Women in Congregate Housing. Journal of Aging Studies.

[R4] Lupik Travis (2014). Downtown Eastside SRO Renovictions Rouse Tenants into Action. The Georgia Straight.

[R5] Mallett Shelley (2004). Understanding Home: A Critical Review of the Literature. The Sociological Review.

[R6] McElroy Justin (2015). Outrage Mounts over Vancouver SRO That’s Been without Heat for Weeks. Global News.

[R7] Robertson Leslie (2007). Taming Space: Drug Use, HIV, and Homemaking in Downtown Eastside Vancouver. Gender, Place and Culture.

[R8] Swanson Jean M, Herman Tamara (2014). On the Brink: The DTES Housing Crisis; 2014 Hotel Survey and Housing Report.

